# Consumption of Feed Supplemented with Oat Beta-Glucan as a Chemopreventive Agent against Colon Cancerogenesis in Rats

**DOI:** 10.3390/nu16081125

**Published:** 2024-04-11

**Authors:** Joanna Harasym, Katarzyna Dziendzikowska, Łukasz Kopiasz, Jacek Wilczak, Rafał Sapierzyński, Joanna Gromadzka-Ostrowska

**Affiliations:** 1 Department of Biotechnology and Food Analysis, Wroclaw University of Economics and Business, 53-345 Wroclaw, Poland; 2 Department of Dietetics, Institute of Human Nutrition Sciences, Warsaw University of Life Sciences, 02-776 Warsaw, Poland; katarzyna_dziendzikowska@sggw.edu.pl (K.D.); joanna_gromadzka-ostrowska@sggw.edu.pl (J.G.-O.); 3 Department of Physiological Sciences, Institute of Veterinary Medicine, Warsaw University of Life Sciences, 02-776 Warsaw, Poland; jacek_wilczak@sggw.edu.pl; 4 Department of Pathology and Veterinary Diagnostic, Institute of Veterinary Medicine, Warsaw University of Life Sciences, 02-776 Warsaw, Poland; rafal_sapierzynski@sggw.edu.pl

**Keywords:** azoxymethane, bioactive compounds, colorectal cancer, oat beta-glucan, oxidative stress, rats, CRC signaling pathway

## Abstract

Colorectal cancer (CRC) accounts for 30% of all cancer cases worldwide and is the second leading cause of cancer-related deaths. CRC develops over a long period of time, and in the early stages, pathological changes can be mitigated through nutritional interventions using bioactive plant compounds. Our study aims to determine the effect of highly purified oat beta-glucan on an animal CRC model. The study was performed on forty-five male Sprague–Dawley rats with azoxymethane-induced early-stage CRC, which consumed feed containing 1% or 3% low molar mass oat beta-glucan (OBG) for 8 weeks. In the large intestine, morphological changes, CRC signaling pathway genes (RT-PCR), and proteins (Western blot, immunohistochemistry) expression were analyzed. Whole blood hematology and blood redox status were also performed. Results indicated that the histologically confirmed CRC condition led to a downregulation of the WNT/β-catenin pathway, along with alterations in oncogenic and tumor suppressor gene expression. However, OBG significantly modulated these effects, with the 3% OBG showing a more pronounced impact. Furthermore, CRC rats exhibited elevated levels of oxidative stress and antioxidant enzyme activity in the blood, along with decreased white blood cell and lymphocyte counts. Consumption of OBG at any dose normalized these parameters. The minimal effect of OBG in the physiological intestine and the high activity in the pathological condition suggest that OBG is both safe and effective in early-stage CRC.

## 1. Introduction

Beta-glucans, a group of polysaccharides, are found in microorganisms like bacteria and yeast, as well as in cereals such as barley and oats. The structural complexity of beta-glucan varies significantly based on its source and the method of isolation, primarily differing in the distribution and length of the side chains. Factors such as primary structure, solubility, degree of branching, and molecular weight play a crucial role in determining the biological activity of beta-glucan [1]. Our previous study showed that dietary administration of beta-glucan isolated from oats exhibited anti-inflammatory, antioxidant, and prebiotic effects in artificially induced enteritis [2]. Furthermore, our results have shown that these effects are related to the molar mass of beta-glucan [2,3], with low molar mass fractions showing more pronounced effects. Additionally, in our recent studies focusing on the effects of oat beta-glucan supplementation on large intestine inflammation (in the absence of early signs of carcinogenesis), we observed that low molar mass oat beta-glucan significantly downregulated the expression of genes associated with pro-inflammatory proteins, as well as reduced the colonic levels of these proteins [3].

It is important to emphasize that in the case of gastrointestinal disorders, especially inflammatory bowel disease or gastrointestinal cancers, multi-component, unpurified oat grain preparations, such as oatmeal or fiber, should not be used as a food ingredient. These preparations contain a variety of active ingredients, including a fraction of insoluble dietary fiber that irritates the intestinal mucosa, as well as proteins that may cause hypersensitivity and/or intolerance. As a more suitable alternative, the use of high purity, low molecular weight 1-3, 1-4-D-beta-glucan as a dietary supplement is recommended. This formulation is devoid of other compounds, including soluble proteins. We intend to use this specific formulation in our *in vivo* CRC animal model. Based on previous research of its anti-tumor activity in *in vitro* studies on skin tumor and lung epithelial cancer cell lines, we anticipate similar effects in this model [4,5].

Colorectal cancer (*colorectal carcinoma*—CRC) is a common malignancy of the gastrointestinal tract, and its incidence is steadily increasing year by year. Epidemiological statistics forecast a rise in the number of new cancer cases and associated deaths in Europe, the United States, and other countries. As a result, colorectal cancer represents a significant global health challenge and ranks as the second most dangerous type of cancer, affecting women and men equally. It is estimated that there were more than 1.9 million new cases of colorectal cancer worldwide in 2020, resulting in approximately 930,000 deaths. The highest occurrence of this cancer type was noted in Australia/New Zealand and various parts of Europe [6]. CRC is also one of the most common cancers in the Polish population. In 2018, it was the third most common malignant neoplasm in men and the second in women. In that year, almost 12,500 deaths due to CRC were recorded in Poland. This type of cancer was also the second most common cause of death from malignant neoplasms in men and the third in women. Despite improvements over the years, 5-year survival rates for colorectal cancer patients in Poland are still among the lowest in Europe [7].

Colorectal cancer primarily originates from the superficial glandular epithelial cells lining the colon or rectum. Aberrant crypt foci (ACF), recognized as preneoplastic lesions of the colon, represent the earliest identifiable intermediate precancerous lesions in CRC. This phenomenon is observed in both laboratory rodents [8,9] and humans [10] and can be identified microscopically on the surface of the entire colonic mucosa, serving as a valuable histological marker in the development of CRC. Furthermore, ACF are also considered as a unique biomarker to evaluate the potential preventive efficacy of various factors or compounds against CRC in the early stages of cancer development [10]. Ezuka et al. (2015) [11], in their comprehensive review, confirmed the potential utility of ACF as a surrogate biomarker for CRC. Additionally, ACF have been used as an endpoint to identify and evaluate the preventive or therapeutic roles of natural and pharmacological compounds, including dietary factors, in CRC development [12,13,14,15]. It is important to note that ACF are a critical juncture at which cancer or tumors may develop. They are readily induced by colon-specific carcinogenesis in rodents, offering a valuable model for studying the process of colorectal cancer development.

Rodents, such as rats, typically exhibit a very low incidence of spontaneous cancer. Therefore, to study colon carcinoma, they are often administered a carcinogen. Azoxymethane (AOM), a chemical mutagen and potent genotoxic agent, is frequently used in these studies. Rodent models of AOM-induced CRC have proven to be highly valuable for investigating the mechanisms underlying carcinogenesis [16]. Additionally, these models are instrumental in studying the preventive effects of various dietary factors [17,18]. The AOM-induced CRC rat model shares many features with human CRC, making it an important model for exploring various aspects of this disease. It has also been found that short-term AOM-induced rat ACF serves as an ideal early biomarker for identifying a range of preventive factors and compounds against CRC [14,19].

Naturally occurring substances of plant origin, particularly polymers, used in animal model experiments are very often unpurified and contain other biologically active compounds, such as polyphenols, protein residues, or other dietary fiber fractions. This raises concerns about the unambiguous assessment of the bioactivity of the tested formulations. Therefore, in our study, we used a chemically pure preparation of oat beta-glucan as an integral component of the rat feed. This preparation was devoid of protein and peptide residues, as well as other components with significant biological potential, such as polyphenols. The main objective of the current study is to assess the effect of a chemically pure fraction of low molar mass oat beta-glucan (OBG), administered *per os* at two doses (1% or 3% *w*/*w*) as a dietary supplement, on the early stages of AOM-induced CRC development in rats. An additional objective is to monitor changes in selected peripheral blood parameters.

## 2. Materials and Methods

### 2.1. Isolation and Characterization of Low Molar Mass Oat Beta-Glucan

A low molar mass oat 1-3, 1-4, beta-D-glucan isolate was prepared from oat bran (Bestpharma, Warszawa, Poland) through a unique, patented process outlined in previous works [20,21]. Initially, the oat bran underwent a freezing process, followed by significant milling while still frozen, repeated several times to effectively diminish particle size. Subsequently, beta-glucan was extracted via alkaline water (pH = 8.5; NaOH). The residual fraction was then separated by centrifugation at 11,000× *g* and discarded. The supernatant was deproteinized at the isoelectric point (pH = 4.5), and the resulting protein precipitate was also removed by centrifugation. A combination of proteolytic, peptidolytic, and amylolytic enzymes was utilized for additional purification, along with enzymatic precipitation of proteins at previously specified isoelectric points [21]. The resulting beta-glucan preparations exhibited 99.3% purity as verified by enzymatic methodology (employing AACC Method 32-23.01, AOAC Method 995.16, AOAC Method 992.28, CODEX Method Type II, EBC Method 3.10.1, ICC Standard No. 166, and RACI Standard Method). These preparations also displayed a molar mass of 5.2 × 10^4^ ± 0.6 × 10^4^ g/mol (52 ± 6 kDa), as confirmed by size exclusion HPLC as described elsewhere [20]. The isolates were analyzed for total polyphenol content using Folin–Ciocalteu reagent [22] and antioxidant activity vs. DPPH, as described previously [23]. Protein content (Lowry method) and total nitrogen (Kjeldahl) were assessed as well.

### 2.2. Animals and In Vivo Experimental Design

The *in vivo* experiment was performed on male outbred Sprague–Dawley rats (n = 45) purchased from Charles River Laboratories (Sulzfeld, Germany). Experimental details were described in our previous publication [24]. Briefly, rats were divided into two main groups: (1) the treatment group (CRC group), in which the early stage of colorectal cancer was chemically induced by peritoneal injection of azoxymethane (AOM) (Sigma-Aldrich, Saint Louis, MO, USA), and (2) the control group, consisting of animals administered the equal volume of 0.9% NaCl solution in the same way as the treatment group. After the last administration of AOM/NaCl, both the CRC and control groups were further divided into 3 dietary subgroups fed during 8 weeks with pellet feed (ZooLab, Sędziszów, Poland) supplemented with different levels of low molar mass oat beta-glucan (OBG), as shown in Table 1. All feeds were formulated based on AIN-93M pellets, which were designed for rats in accordance with the species-specific nutritional recommendations of Reeves et al. (1993) [25]. Body weight was recorded on a weekly basis, and feed intake was monitored every day. Clinical status indicators (including appetite loss, body weight loss of more than 10%, loose and bloody stools, diarrhea, anus edema, and rectal bleeding) were monitored twice daily throughout the experiment.

All experimental procedures were conducted with the approval of the 2nd Local Ethical Committee in Warsaw, Poland (resolution No. WAW2/040/2019, 15 March 2019) in accordance with the UE Directive (2010/63/UE), Polish law and the principles of 3R rules (Replacement, Reduction, and Refinement). According to the 3R rules, the number of rats in the study subgroups is the smallest possible from the point of view of statistical verification of the results and was determined based on the scientific literature [26] using available tools for estimating the number of animals in the experimental groups (http://biomath.info/power/ttest.htm; accessed on 3 March 2019).

### 2.3. Blood and Tissues Sampling

After 8 weeks of feeding, the rats were bled by cardiac puncture under Izoflurane (Aerrane Isoflurane USP, Baxter, Poland) general anesthesia. Subsequently, the entire large intestine of each rat was collected. A piece from each of the three parts of the large intestine (cecum, colon, and rectum) was collected and frozen together in liquid nitrogen for Western blot analysis or fixed in 10% buffered formalin, designed for histopathology, histomorphometry, and immunohistochemistry analysis. The other parts were immediately frozen in liquid nitrogen and stored at −80 °C until biochemical analysis.

### 2.4. Hematological Analysis and Blood Plasma Separation

Peripheral blood collected from the heart was separated into two portions: one for whole blood and the other for plasma. The whole blood sample was subjected to hematological analysis using the Abacus Junior Vet analyzer (Diatron, Budapest, Hungary). This analysis included the determination of the total number of white blood cells (WBC), monocytes and eosinophils (MID), lymphocytes (LYM), granulocytes (GRA), red blood cells (RBC), and platelets (PLT). Additionally, mean corpuscular volume (MCV), red cell distribution width (RDW), mean corpuscular hemoglobin concentration (MCHC), and mean corpuscular hemoglobin (MCH) in cells, as well as hemoglobin concentration (HGB) and hematocrit (HCT) of blood were measured. Blood plasma was obtained by centrifugation of whole blood at 2200× *g* for 10 min at 4 °C. The obtained plasma samples were stored at −20 °C until biochemical analyses were performed.

### 2.5. Histopathological and Histomorphometry Evaluation

The collected biological samples of the large intestine specimens were fixed in 10% buffered formaldehyde, subsequently dehydrated through graded ethanol and xylene baths, and then embedded in paraffin wax. Sections of 4 µm thickness were prepared and stained with hematoxylin and eosin (H&E) for examination. The analyses were examined using a standard light microscope Olympus BX41 (Olympus, Tokyo, Japan) according to histological criteria of multistep carcinogenesis as proposed by Perse and Cerar (2011) [14]. The early stage of carcinogenesis was characterized by recognizable histological changes, beginning with aberrant crypt foci (ACF), which were identified by a veterinary histopathologist. Mucosal lesions were classified into various categories: normal mucosa, hyperplastic crypts, dysplastic crypts, adenoma, and carcinoma.

In addition, a histomorphometric examination of colonic crypts, including their length and width, was performed to provide a more detailed characterization of changes in the early stages of carcinogenesis. The samples selected for histomorphometry were those of the highest quality. Histomorphometric analyses were conducted using an Olympus BX41 microscope (Olympus, Tokyo, Japan) coupled to a computer equipped with the CellA^®^ analysis system. For each colon specimen, areas with clearly visible and well-stained crypts with visible histologic elements were selected. In each sample, 10 to 30 crypts were analyzed. The software was used to measure the length of the crypt and its width in the upper, middle, and lower thirds of the length.

### 2.6. Blood Plasma Biochemical Parameters

The blood cell lysate used to assess antioxidant enzyme activity was prepared according to the protocol described in the instructions provided with the Randox kit (Randox in County Antrim, UK). The blood cell lysate was used for the analysis of total antioxidant status (TAS) and thiobarbituric acid reactive substances concentration (TBARS), as well as glutathione reductase (GR), glutathione peroxidase (GPx), and superoxide dismutase (SOD) activity. TAS, GR, GPx, and SOD were determined using the commercial Randox assay kits (TAS, Glutathione Reductase, RANSEL, and RANSOD kit; Randox Laboratories Ltd., Crumlin, Co. Antrim, UK) according to the manufacturer’s protocols. The catalog numbers of the kits were as follows (TAS, cat. no.: NX2332; GR, cat. no.: GR2368; GPx, cat. no.: RS504/505/506; SOD, cat. no.: SD125). Thiobarbituric acid reactive substances (TBARS) were analyzed according to the method described by Aguilar Diaz De Leon and Borges (2020) [27].

### 2.7. Methods for RNA Isolation, Conversion to cDNA, and Gene Expression Analysis

RNA was extracted from large intestine samples using the RNeasy Lipid Tissue Mini Kit (Qiagen, Hilden, Germany) according to the supplied protocol. The purity and concentration of the extracted RNA were determined using the NanoDrop™ 2000 spectrophotometer (Thermo Fisher Scientific, Waltham, MA, USA). To assess the integrity of the RNA, the Agilent Bioanalyzer 2100 system was used in conjunction with the RNA 6000 Nano LabChip^®^ Kit (Agilent Technologies, Palo Alto, CA, USA) on randomly selected RNA samples. This assessment revealed a low rate of RNA degradation as evidenced by an RNA integrity number (RIN) exceeding 9. The RT2 First Strand Kit (Qiagen, Hilden, Germany) was then used to synthesize complementary DNA. For gene expression analysis focusing on specific pathways, the Custom RT2 Profiler™ PCR array, provided by Qiagen, was applied according to the manufacturer’s guidelines. This analysis was performed in two technical replicates for each colon sample to ensure the accuracy and reliability of the results. The RT2 Profiler PCR arrays (Qiagen, Hilden, Germany) contain primers for a curated analysis of genes essential for the mechanisms of carcinogenesis induced by AOM. These include *Ctnnb1* (catalog number PPR57457A), *Wnt1* (catalog number PPR57724A), *Apc* (catalog number PPR52699A), *Dclk1* (catalog number PPR52232A), and *Smad4* (catalog number PPR06574C). Amplification was performed using the AriaMx Real-time PCR System (Agilent Technologies, Palo Alto, CA, USA), starting with an initial denaturation at 95 °C for 10 min. This was followed by 40 amplification cycles, each including 15 s of denaturation at 95 °C and 1 min of annealing/extension at 60 °C. The threshold cycle (Ct) method was used to quantify relative gene expression using *B2m* (catalog number PPR42607A) and *Ldha* (catalog number PPR56603B) as reference genes for normalization. Analysis and calculation of results were facilitated by GeneGlobe Qiagen Software (https://geneglobe.qiagen.com/pl, accessed on 3 April 2023). The expression levels of the target genes, normalized to *B2m* and *Ldha*, are presented as fold changes relative to a control group, which is established at a baseline value of 1 to illustrate changes in expression.

### 2.8. Immunohistochemical Analysis

Immunohistochemical analysis was performed as described by Kopiasz et al. (2024) [24]. Briefly, tissue slices (5 µm thickness) containing three representative pieces of the large intestine from each rat were deparaffinized in xylene and rehydrated in a series of decreasing concentrations of ethanol. After recovering antigens and blocking endogenous enzymes, the samples were incubated with primary antibodies overnight at 4 °C using a rabbit anti-β-Catenin polyclonal antibody (Cat#8480, 1:200, Cell Signaling Technology, Danvers, MA, USA). The samples were then labeled with polymers consisting of anti-rabbit antibodies conjugated to the horseradish peroxidase (HRP) enzyme complex. 3,3′-diaminobenzidine (DAB) was used to obtain a brown color, and hematoxylin was used to counterstain nuclei. The immunohistochemically stained slides were examined using a NIKON Eclipse Ti2 microscope (Nikon, Melville, NY, USA—funding details in Supplementary Data). On the recorded images, six mucosal areas were marked for each of the three sections of the colorectum. A total of eighteen marked mucosal areas for the colorectum from one rat were analyzed together. Colorimetric saturation (brown colors reflecting antigen expression) and object area were measured using the NIS-Elements BR 5.01 program.

### 2.9. Western Blot Analysis

Western blot analysis was conducted following the previously described protocol [24]. Briefly, samples from three different parts of the large intestine from each rat were homogenized together in RIPA buffer supplemented with a cocktail of phosphatase and protease inhibitors. The lysates were then centrifuged, and the supernatants were collected. The samples (normalized to a 50 µg protein concentration) were resolved by sodium dodecyl sulfate-polyacrylamide gel electrophoresis and transferred onto a PVDF membrane. Next, the membranes were incubated overnight at 4 °C with primary antibodies, including rabbit anti-β-Catenin monoclonal antibody (Cat#A19657, 1:1250, ABclonal, Woburn, MA, USA) and mouse anti-β-actin (8H10D10) monoclonal antibody (Cat#3700, 1:5000, Cell Signaling Technology, Danvers, MA, USA), under gentle shaking. After that, the membranes were incubated with secondary antibodies conjugated with IR fluorophore (IRDye^®^ 800 CW anti-rabbit antibody) at 1:5000 dilution. The protein expression was analyzed using the ChemiDoc Imaging System (Bio-Rad, Hercules, CA, USA). The integrated optical density (IOD) was quantified using the Image Lab 6.1 Software (Bio-Rad, Hercules, CA, USA). The relative levels of the analyzed proteins were normalized to β-actin.

### 2.10. Statistical Analysis

The collected data were analyzed using Statistica software (version 13.3 PL; StatSoft, Krakow, Poland). Prior to further analyses, the equality of variance and normality of distribution were determined for all datasets. The results of MID required a square root transformation to achieve normal distribution and equality of variance. Two-way analysis of variance (ANOVA) was used to evaluate the effect of two experimental factors—the early stage of CRC and the type of dietary intervention—as well as their interaction. Tukey’s post hoc test was used to determine the significance of outcome differences among the groups. Differences between the control group (control OBG_0) and the other groups were analyzed using Dunnett’s post hoc test. Pearson’s chi-square test with Cramer’s V value was used to analyze the results of histopathological changes. Statistical significance was established at a *p*-value of less than 0.05. All graphs were generated using GraphPad Prism, version 9.3.1, from GraphPad Software (GraphPad Software Inc., San Diego, CA, USA).

## 3. Results

### 3.1. Chemical Characterization of Low Molar Mass Oat Beta-Glucan

The detailed characteristics of the oat beta-glucan isolates used in the present study are comprehensively presented in Table 2. The extensive process of extraction, followed by isoelectric precipitation, effectively removes the majority of proteins. Subsequent dissolution and organic solvent precipitation denatures and hydrolyzes any proteinaceous residues, as well as enzymes used [21]. Exhaustive extraction and purification are performed to remove any potentially bioactive molecules, which could bias the assessment of oat beta-glucan impact. Small molecule antioxidant compounds such as polyphenols can modulate the system response to an administered substance. The tested sample of oat beta-glucan isolate was analyzed for total polyphenols content and antioxidative activity vs. DPPH radical, revealing negligible activity and proving to be free from these two bioactive contaminants. The bioactivity of beta-glucan depends not only on its purity but also on the molar mass of the analyzed fractions. The average molar mass of the isolate was determined using internal standards of oat beta-glucan, resulting in 5.2 × 10^4^ ± 0.6 × 10^4^ g/mol.

### 3.2. Feeds Nutritional Characteristic, Feed Intake, and Body Weight Gain

In our study, we used a chemically pure beta-glucan from oats as a part of rat feed in early-stage colon cancer models. The beta-glucan was free of proteins, peptides, polyphenols, and other biologically active compounds. The purity of this polysaccharide allowed for an accurate evaluation of the therapeutic effects of beta-glucan without the risk of additional intestinal irritation or hypersensitivity. Our choice of low molar mass beta-glucan was based on its proven anti-inflammatory and indirect antioxidant properties in previous studies [2,3]. OBG was added to the feed at 1% and 3% levels, substituting for equivalent carbohydrate content, maintaining the feed’s balance without altering the insoluble fiber fraction. Two doses were used based on dose conversion from human to animal and vice versa, taking into account body surface area, metabolic rate, and physiological processes. Using appropriate formulas [28], the consumption of feed supplemented with the addition of OBG at 1% *w*/*w* in rats is equivalent to consuming approximately 8–10 g OBG for an adult human (BW 60–70 kg), while 3% corresponds to approximately 24–30 g. Furthermore, a dose of 3% was administered to observe any potential dose-dependent effects.

The semi-synthetic purified feeds (pellets) used in the present study were formulated using natural products (corn starch, maltodextrin, sucrose, α-cellulose, casein, and soybean oil) as detailed in Supplementary Table S1. The declared and analyzed compositions of these experimental and control feeds, including proteins, carbohydrates, fat, and fibers, are shown in Supplementary Table S2. The content of 1-3, 1-4, beta-D-glucan in the feeds was determined according to AOAC 995.16 method (K-BGLU, Megazyme, Ireland) based on lichenase digestion. Additionally, the metabolizable energy (ME) of the feeds and the feed growth efficiency (Table 3) were also calculated using the following formulas in accordance with Bielohuby et al. (2010) [29].

ME (MJ/kg) = 0.0223 × protein (g) + 0.0341 × fat (g) + 0.017 × starch (g) + 0.0168 × sugar (g) + 0.0074 × (organic matter − protein − fat − starch − sugar − fiber) (g) − 0.0109 × fiber (g)(1)

Feed growth efficiency = total weight gain (g)/the calculated amount of energy intake with the feed (MJ)(2)

**Figure 1:**
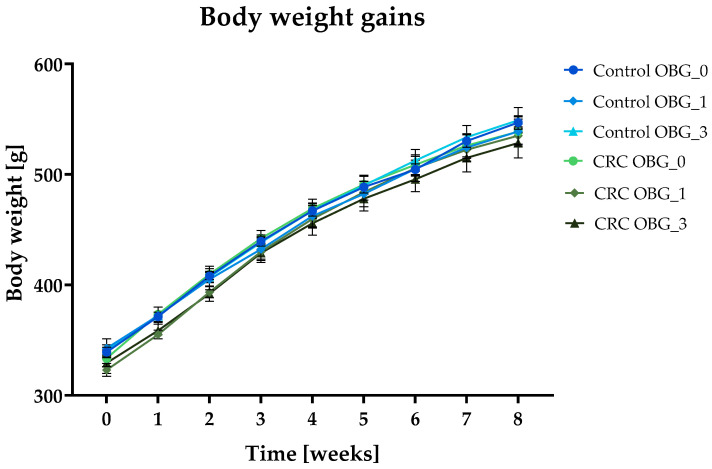
Body weight gain curves during 8 weeks of experiments [mean ± SE].

The nutrient content of the experimental feeds, as declared by the manufacturer, aligns with the recommended levels for adult rats. The analytically determined beta-glucan content in the feeds did not differ from the assumed level in the experiment. Additionally, the growth efficiency of all feeds used in the experiment showed no significant differences. This uniformity in feed quality led to consistent feed intake across all rat groups, with no notable disparities in body weight gain. The body weight gain of all groups of rats followed a similar trajectory throughout the study, as shown in Figure 1.

### 3.3. Clinical Signs, Necropsy Observations, and Macroscopic Large Intestine Evaluation

During the 8-week period following the AOM peritoneal injection, the rats were monitored twice daily for several health indicators, including appetite, morbidity, stool consistency, and the appearance of the anus. These clinical observations did not reveal any signs indicative of poor health, such as lack of appetite, abnormal appearance of the anus, hair loss, or diarrhea. Similarly, during necropsy, no pathological changes were observed upon visual examination of the large intestine.

### 3.4. Histopathological Changes in the Large Intestine

**Figure 2:**
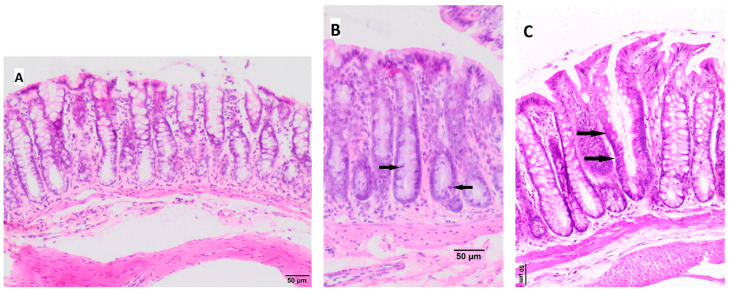
Histopathological changes in the large intestine mucosa. (**A**)—normal mucosa, (**B**)—hyperplastic crypts with an increased number of epithelial cell nuclei (crowded nuclei), lower number of goblet cells, and increased mitotic activity (some mitotic figures indicated by black arrows) restricted to the lower two-thirds of the crypts, (**C**)—one aberrant crypt (black arrows) is present among normal crypts—slightly enlarged crypt diameter and low number of goblet cells are visible. Hematoxylin–eosin staining, magnification: (**A**)—100×, (**B**)—200×, (**C**)—200×.

Histopathological examination assessed the appearance of intestinal crypts for the presence of aberrant crypt foci (ACF), which may be hyperplasia/dysplasia/adenoma/cancer, as shown in Figure 2. In the Control OBG_0 group and Control OBG_1 group, no morphological changes of crypts were observed; in one rat from the Control OBG_3 group, the presence of single hyperplastic crypts was observed. In the CRC groups, hyperplastic crypts were observed mostly in group CRC_OBG_0 and less commonly in groups CRC OBG_1 and CRC OBG_3. Detailed data on the occurrence of hyperplastic lesions in intestinal crypts of particular groups of rats are presented in Table 4 and Supplementary Table S3.

No significant differences were observed in the measurements of the crypt length and width at the bottom, upper and middle parts of the crypts between the CRC and control groups, as detailed in Table 5.

### 3.5. Red Blood Cells and Platelet Parameters

As shown in Table 6, analysis of variance for RBC, HGB, HCT, MCV, MCH levels, and PLT counts revealed no significant differences between the study groups. However, rats from the CRC group that consumed the feed supplemented with 3% OBG had higher MCHC values compared to the control OBG_3 (*p* < 0.05) and OBG_0 (*p* < 0.01) groups. Additionally, significant differences (*p* < 0.05) in RDWc values were observed between the control group (OBG_0) and the CRC-induced group fed with 3% OGB (OBG_3).

### 3.6. White Blood Cells Parameters

**Figure 3:**
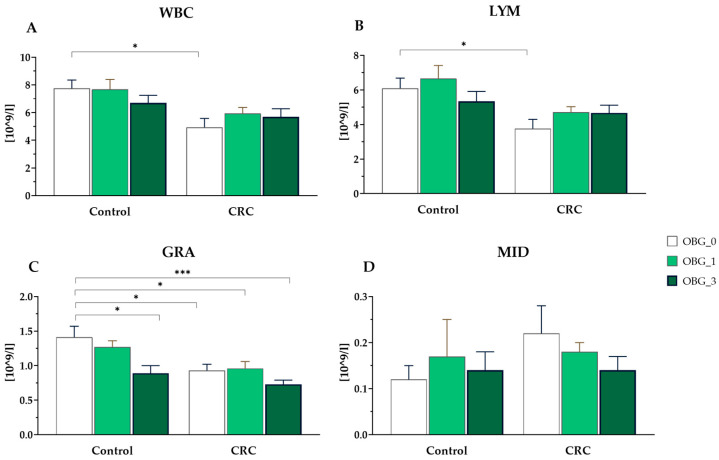
White blood cell parameters (mean ± SE). (**A**)—total white blood cell count; (**B**)—lymphocyte count; (**C**)—granulocyte count; (**D**)—total number of the other types of white blood cells not classified as lymphocytes or granulocytes. Significantly different from the control group (OBG_0) (* *p* < 0.05; *** *p* < 0.001) (Dunnett’s post hoc test).

The total white blood cell and lymphocyte counts were lower in rats from the CRC OBG_0 group compared to the control OBG_0 group (*p* < 0.01). However, there was no significant difference in the total number of other types of leukocytes among all groups. Notably, the most significant differences between groups were found in the number of granulocytes. In the control rats, consumption of feed with 3% OBG resulted in a significant reduction in the number of granulocytes (*p* < 0.05). A similar difference was observed in the CRC rats fed with feed containing 3% OBG compared to the control group (*p* < 0.001) (Figure 3).

### 3.7. Peripheral Blood Plasma Redox Status Parameters

**Figure 4:**
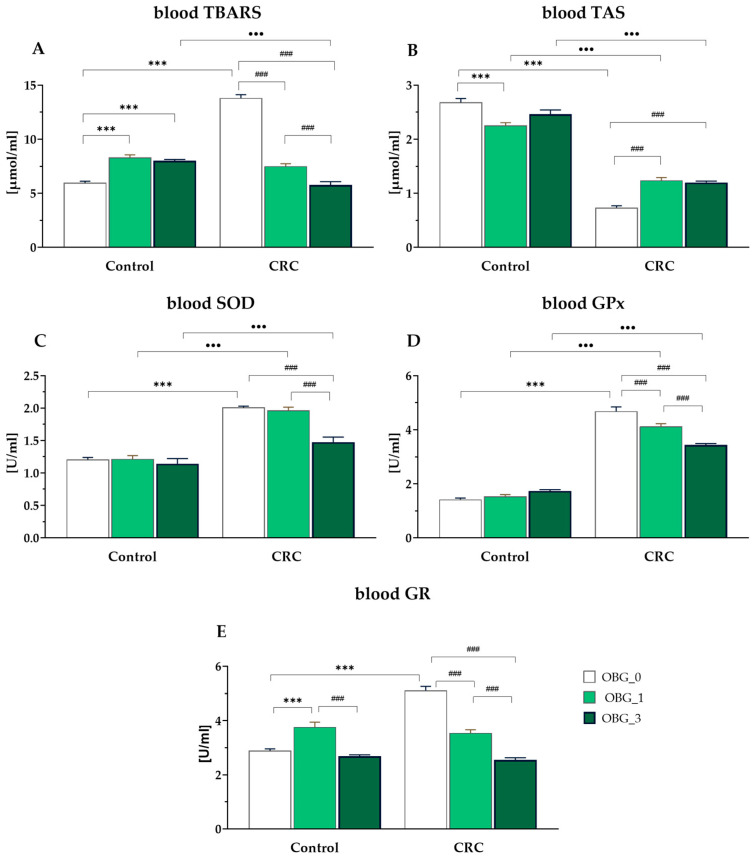
Peripheral blood redox status parameters in CRC and control rats (mean ± SE): (**A**)—thiobarbituric acid-reactive substances (TBARS). (**B**)—total antioxidant status (TAS); (**C**)—superoxide dismutase (SOD); (**D**)—glutathione peroxidase (GPx); (**E**)—glutathione reductase (GR); Significantly different from the control group (OBG_0) (*** *p* < 0.001) (Dunnett’s post hoc test). Significant differences within control and CRC groups between dietary subgroups (### *p* < 0.001) (Tukey’s post hoc test). Significant differences between the respective control and CRC groups on the same feed (^●●●^ *p* < 0.001) (Tukey’s post hoc test).

Thiobarbituric acid reactive substances (TBARS) are a by-product of lipid peroxidation and are recognized as a marker of accelerated lipid oxidation. In the present study, an increased accumulation of TBARS was detected in the systematic circulation of CRC rats consuming feed without OBG supplementation, with significantly higher values compared to other CRC and control groups (*p* < 0.0001). The eight-week dietary intervention with OBG significantly reduced TBARS levels, with the most significant reduction noted in the OBG_3 group. In the control groups, TBARS levels were higher in the OBG_1 and OBG_3 groups compared to the values of this parameter in the OBG_0 group (Figure 4A). Total antioxidant status (TAS) of peripheral blood was significantly lower in the CRC rats fed with feed without OBG compared to other CRC groups, regardless of the level of OBG supplementation, and to all control groups (*p* < 0.0001). Specifically, in the control rats, a significantly lower TAS was observed in the OBG_1 group compared to the OBG_0 group, as shown in Figure 4B. There was also a significant increase in the activity of antioxidant defense enzymes (GPx and SOD) in CRC animals fed with feed without OGB compared to control animals fed the same feed (*p* < 0.0001). SOD activity in rats from the CRC OBG_3 group was significantly decreased compared to the CRC OBG_0 group and did not differ from the control groups (Figure 4C). In addition, blood GPx activity in the CRC rats from the OBG_0 group was significantly higher than that observed in the OBG_1 and OBG_3 groups (*p* < 0.001 and *p* < 0.0001, respectively). In control animals, GPx activity did not differ among the nutritional subgroups (Figure 4D). In the blood of CRC rats, GR activity was found to be higher in the OBG_0 group compared to the OBG_1 and OBG_3 groups (*p* < 0.001 for all comparisons). Conversely, in the control rats, the highest GR activity was observed in the OBG_1 group (*p* < 0.001) (Figure 4E).

### 3.8. CRC Signaling Pathways

**Figure 5:**
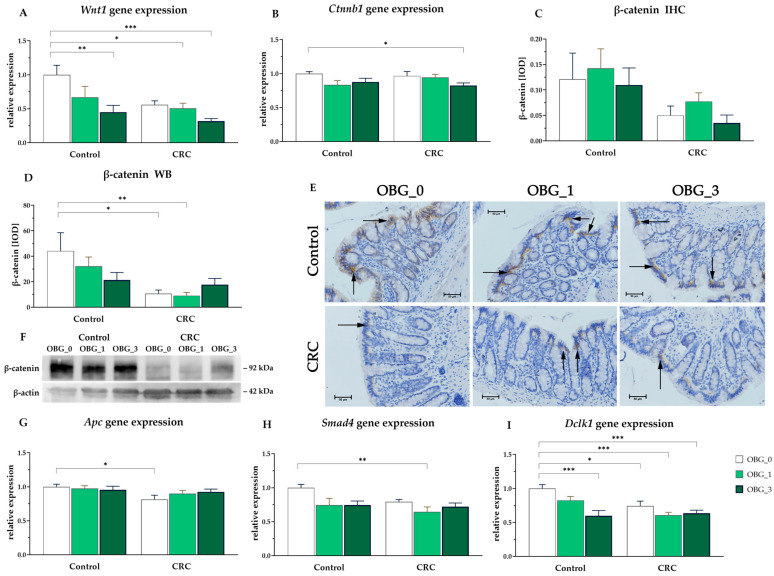
CRC signaling pathways. (**A**,**B**,**G**–**I**)—Changes in relative gene expression in the large intestine of rats: (**A**)—*Wnt1* (Wnt family member 1); (**B**)—*Ctnnb1* (β-catenin); (**G**)—*Apc* (Adenomatous polyposis coli); (**H**)—*Smad4* (Mothers against decapentaplegic homolog 4); (**I**)—*Dclk1* (Doublecortin-like kinase 1). Data are presented in arbitrary units as a ratio of the expression of the target gene to the mean expression of the reference genes (*B2m* and *Ldha*) with the control group calculated as 1. (**C**–**F**)—Changes in β-catenin protein expression; (**C**)—in the large intestine mucosa using immunohistochemistry analysis (IHC) presented as integrated optical density (IOD), (mean ± SE); (**D**)—in the whole large intestine wall presented as IOD, (mean ± SE); (**E**)—Light micrographs imaged (×400 magnification). Black arrows indicate areas with high expression of β-catenin (brown precipitate); (**F**)—Representative immunoblot images. Significantly different from the control group (OBG_0) (* *p* < 0.05, ** *p* < 0.01, *** *p* < 0.001) (Dunnett’s post hoc test).

The gene expression results associated with the WNT/β-catenin signaling pathway, which is implicated in CRC tumorigenesis, showed a significant effect of both OBG and rat health status on the modulation of *Wnt1* expression (ANOVA, *p* < 0.01 and *p* < 0.05, respectively, Figure 5A). *Wnt1* gene expression was downregulated in rats from the CRC groups, as well as in animals fed with feed supplemented with OBG. Post hoc analysis revealed that OBG supplementation decreased *Wnt1* gene expression at the highest OBG concentration (3%) in both control and CRC groups (*p* < 0.05). Additionally, in the CRC OBG_1 group, *Wnt1* gene expression was significantly lower compared to control rats receiving feed without OBG.

The expression of β-catenin (*Ctnnb1*), a crucial component of the Wnt/β-catenin signaling pathway implicated in cell proliferation and tumorigenesis, was examined at both the gene and protein levels. *Ctnnb1* mRNA expression in colorectal tissue is shown in Figure 5B. A significant effect of OBG supplementation on the expression of *Ctnnb1* was observed (ANOVA, *p* < 0.05). Significant downregulation of *Ctnnb1* was observed in the CRC group supplemented with the highest level of OBG (CRC OBG_3) compared to the control group without OBG supplementation (control OBG_0) (*p* < 0.05). Significant effects on β-catenin protein expression were found only by Western blot analysis (in the entire large intestine wall). Induced early-stage carcinogenesis significantly decreased the expression of this protein (ANOVA, *p* < 0.01), whereas, in the CRC OBG_3 group, β-catenin expression was restored to levels comparable to those observed in the control groups (Figure 5D,F). The expression of this protein in the mucosa did not significantly differ between the study subgroups (Figure 5C,E). However, two-way ANOVA revealed that the early stage of cancerogenesis decreased the expression of this protein (ANOVA, *p* < 0.01).

Two-way ANOVA revealed that the induction of early-stage CRC significantly affected the downregulation of the *Apc* gene (Figure 5G), which is a critical regulator in the WNT signaling pathway and a known tumor suppressor gene, as well as the *Smad4* gene (Figure 5H), also a suppressor gene (ANOVA, *p* < 0.05). Post hoc analysis revealed a significant downregulation of *Apc* expression in the CRC group without OBG supplementation (CRC OBG_0) compared to the control group also without OBG (Control OBG_0) (*p* < 0.05). Interestingly, supplementation with oat beta-glucan mitigated the adverse effects of CRC on *Apc* expression. In the CRC groups receiving OBG, *Apc* expression levels were restored to levels comparable to those observed in the control groups. Furthermore, two-way ANOVA revealed significant effects of both CRC-induced and OBG supplementation on *Smad4* gene expression (ANOVA, *p* < 0.05). In particular, a distinct downregulation of *Smad4* expression was revealed in CRC OBG_1 compared to the control OBG_0 group. The *Smad4* gene was downregulated by OBG. However, the inclusion of 3% OBG in the feed of CRC-affected rats restored *Smad4* expression levels to those comparable to the corresponding control group. The expression of the oncogenic *Dclk1* gene (Figure 5I) was influenced by all experimental factors and their interactions (ANOVA, *p* < 0.05). Post hoc analysis revealed that *Dclk1* gene expression was lower in rats from the CRC groups fed with the control feed compared to the control group without OBG supplementation (control OBG_0) (*p* < 0.05). Additionally, *Dclk1* gene expression was lower in the CRC OBG_1, CRC OBG_3, and control OBG_3 groups compared to the control OBG_0 (*p* < 0.05).

## 4. Discussion

In our 8-week study, we focused on the impact of OBG supplementation on the rat CRC model with AOM-induced carcinogenesis. Throughout the study period, rats were observed for health indicators, including appetite and physical appearance, with no adverse signs noted in those fed with OBG-supplemented feed. In contrast, CRC rats exhibited early signs of carcinogenesis, such as ACF in the mucosal layer, as well as its erosion and hyperplasia. Blood analysis across different groups showed no major differences in most hematological values, but there were slight MCHC and RDWc changes in CRC rats fed with 3% OBG, alongside notable disparities in white blood cells and granulocytes, indicating an immune response induction. The induction of CRC resulted in a significant increase in the concentration of the oxidative stress marker (TBARS level) and a decrease in antioxidant defense, as evidenced by a significant reduction in total antioxidant status (TAS). This was accompanied by an increase in endogenous antioxidant enzymes, such as superoxide dismutase (SOD) and glutathione peroxidase (GPx). Furthermore, OBG supplementation reduced the TBARS level and enhanced the antioxidant defense, as evidenced by the increased TAS and decreased activities of antioxidant enzymes such as GPx and SOD. This suggests a protective role of OBG against oxidative damage. The analysis of gene and protein expression revealed the effect of OBG supplementation and CRC status on the modulation of the WNT/β-catenin signaling pathway. This pathway is crucial in cell proliferation and tumorigenesis. OBG appeared to mitigate the adverse effects of CRC on this pathway, suggesting its potential as a therapeutic agent for CRC management.

Colorectal cancer (CRC) develops from the superficial glandular epithelial cells lining the colon or rectum through a multistep process known as carcinogenesis [30]. Aberrant crypt foci (ACF) are recognized as pre-neoplastic lesions in the colon and represent the earliest microscopically identifiable intermediate precancerous lesions during colon carcinogenesis in both laboratory rodents [31,32,33] and human populations [34]. The identification of ACF as pre-neoplastic lesions in the colon underscores their significance in CRC research. Numerous studies have used ACF as a surrogate endpoint for chemoprevention in rats, illustrating their value in early detection and prevention strategies for CRC [10,35]. ACF are recognized as a valuable histological biomarker that indicates the development stage of colorectal carcinogenesis [36]. They have been used to identify and evaluate the preventive or therapeutic role of various natural and pharmacological compounds, including dietary factors, in colorectal cancer development [37]. The size and density of ACF can increase over time, with significantly higher densities noted in the later stages of CRC compared to the early stages, highlighting their potential as indicators of disease progression [36]. In our study, colorectal ACF were present in some rats, indicating very early stages of CRC development, which is also confirmed by other studies [38,39]. Although they were few in number, these ACF signify the initiated process of carcinogenesis.

The classical diagnosis of CRC in humans primarily relies on colonoscopy, followed by examination of biopsied tissue using hematoxylin and eosin staining. While considered the gold standard in the CRC diagnosis, this assessment can be somewhat subjective, relying on the expertise of pathologists, particularly in ambiguous cases. However, the integration of artificial intelligence (AI) and other technological innovations into diagnosis is improving both its accuracy and efficiency [40]. To improve the precision of diagnosis, novel approaches such as histomorphometry are being developed to provide an unbiased evaluation of ACF. This method offers both qualitative and quantitative analysis of tissue structures, providing insights that might be ambiguous to the pathologist, thereby facilitating standardized examination and objective diagnosis. In our study, histological examinations successfully identified the presence of ACF, a key early marker in the development of CRC. However, the histomorphometric analysis did not reveal any significant changes in the crypt parameters. This discrepancy suggests that while histology is sensitive in detecting early morphological alterations associated with ACF, histomorphometry in our study did not provide additional insights into these early lesions. The lack of detectable histomorphometric changes despite the histologic presence of ACF may reflect the subtle nature of these changes during the early stages of CRC development, which are below the detection threshold of histomorphometric techniques.

The AOM-induced colon cancer model in laboratory rodents is frequently used to study the underlying mechanisms of human sporadic colorectal cancer formation. This is because the tumor development mechanism in animals is very similar to that observed in humans. Intraperitoneal administration of AOM causes ACF, leading to the development of malignant adenoma. AOM does not directly interact with DNA and must be activated *in vivo* to promote carcinogenesis. Azoxymethane is metabolized by cytochrome P450, specifically by its CYP2E1 isoform. The first stage of this transformation is the hydroxylation of the AOM methyl group to form methylazoxymethanol (MAM) and its subsequent decomposition into formaldehyde and a highly reactive methyl diazonium. This chemical substance causes DNA mutation, which can initiate the tumorigenesis process by acting on several key genes in intracellular signaling pathways [16].

Disturbances in redox status play a significant role in the actions of AOM and the associated risk of CRC formation and development. This association between oxidative stress and CRC has also been confirmed by various studies in the human population [41,42,43,44] as well as by findings from experiments using animal models of chemically induced CRC [45,46]. Redox imbalance primarily results in an increase in oxidative stress, which involves the excessive generation and accumulation of free radicals in cells. Oxidative stress plays a critical role in cellular damage and mutation, contributing to the initiation and progression of CRC [47,48].

Excessive accumulation of free radicals in the cells of the intestinal mucosa causes oxidative stress, which significantly alters the immune response of this part of the gastrointestinal tract. These alterations lead to metabolic changes that are critical for the initiation of cancer formation through multiple pathways [49,50]. These pathways can impair biological membranes by oxidizing their constituent lipids, cause DNA oxidative damage within the nucleus, and oxidize proteins and carbohydrates among the chemical components of the cell. The byproducts of these pathways can act as potentially destructive factors during the initiation and development of CRC [51]. In our current study, we found that the plasma concentration of TBARS was significantly higher in the CRC rats compared to the control animals. Additionally, this alteration was accompanied by an increase in the activity of antioxidant enzymes such as SOD, GPx, and GR, which are natural protective mechanisms that counteract the destructive effects of oxidative stress [47]. The activities of SOD and GPx in peripheral blood are reliable biomarkers of oxidative stress used in CRC patients [52], as well as in *in vivo* studies [53]. Antioxidant enzymes, including SOD, serve as the primary line of endogenous antioxidant defense and are highly susceptible to oxidative damage from carcinogens. Agents that counteract the action of carcinogens suppress the activity of antioxidant enzymes and reduce the overproduction of free radicals, thereby restoring the redox balance [54]. Supplementation with OBG significantly decreased TBARS levels and the activity of GPx, GR, and SOD in animals with CRC. Our previous study showed similar results in animals with TNBS-induced colitis, demonstrating that low molar mass OBG fractions could reduce oxidative stress and inflammation [55]. In the present study, we used OBG, which lacks antioxidant activity. This suggests that its effect on the redox status of CRC rats was indirect. The proposed mechanism of OBG’s indirect action may involve the phosphoinositide-3-kinase-protein kinase B/Akt (PI3K-Akt) signal transduction pathway. Furthermore, water-soluble non-starch polysaccharides may also stimulate the insulin receptor α (IRα)-mediated PI3K-Akt signaling pathway to exert antioxidant activity [56].

The results of our study indicate that the hematological parameters, including red blood cell (RBC) counts, hemoglobin (HGB) levels, hematocrit (HCT) percentages, mean corpuscular volume (MCV), mean corpuscular hemoglobin (MCH), and platelet counts, remained consistent among the different experimental groups. This suggests that the induction of early-stage CRC and the dietary intervention with an OBG feed did not significantly alter these parameters. These results are consistent with research on patients with colorectal cancer, where changes in RBC parameters were found in the later stages of this cancer [57]. Based on a large meta-analysis, the authors suggest that RBC parameters, as well as WBC and platelet counts, can be used as predictors for referral to clinical testing for cancer diagnosis in the later stages of CRC [58]. However, as was shown by our results, a notable exception was observed in the MCHC level, which was significantly higher in rats from the CRC group consuming the feed supplemented with 3% OBG, indicating a specific response to dietary intervention at the cellular level that warrants further investigation for its potential implications in CRC pathophysiology.

During tumorigenesis in the early stages of colorectal cancer, slight changes in hematologic parameters may occur. These changes can provide important insight into the body’s response to the developing cancer. For example, an increase in RDWc values, associated with higher variability in erythrocyte size, is a condition that can be caused by several factors, including inflammation and nutritional deficiencies, which are common in CRC patients [59,60]. Variations in RDWc are particularly significant as they may reflect the early systemic impact of CRC. Therefore, monitoring these parameters could provide valuable prognostic information in the early stages of colorectal cancer [61].

The study observed a reduction in WBC, GRA, and LYM in rats from the CRC OBG_0 group compared to the control group, indicating a potential immunomodulatory effect of the CRC condition. A decrease in WBC, GRA, and LYM levels in CRC animals could potentially indicate a compromised immune response, which is crucial in the body’s defense against cancer progression. Lymphocytes, including T and NK cells, play a crucial role in antitumor immunity. A decrease in lymphocyte count may indicate impaired immune system function, which can promote tumor cell survival and progression [62,63]. Notably, the significant decrease in GRA counts in control rats fed 3% OBG feed compared to the control group highlights the anti-inflammatory potential of the OBG feed. This suggests that dietary OBG may play a role in modulating immune responses.

CRC is a complex disease characterized by the dysregulation of multiple signaling pathways, among which the WNT/β-catenin pathway plays a pivotal role in tumorigenesis. This pathway is crucial for maintaining the balance between cell proliferation and apoptosis, which is essential for intestinal homeostasis and regeneration [64]. The sequence of sporadic events leading to the development of CRC is still not sufficiently well understood. Most evidence suggests that the initiating event of CRC formation is hyperactivation of the WNT signaling pathway, primarily through mutations in β-catenin or the APC gene. Mutations in key components of the WNT/β-catenin pathway, such as APC and β-catenin, are frequently observed in CRC patients, further underscoring the significance of this pathway in CRC development. Abnormalities in the WNT pathway occur in most patients with sporadic CRC. More than 80% of adenomas exhibit APC mutations, and an additional 5–10% have mutations or epigenetic changes in other parts of the WNT signaling pathway, such as β-catenin. It has been demonstrated that azoxymethane causes mutations in β-catenin at codons 33 and 41, leading to the accumulation of β-catenin in the carcinogenesis process [16]. The results of the present study indicate that OBG supplementation modulates the WNT/β-catenin pathway, which is a key pathway in the development and progression of CRC. CRC condition and oat beta-glucan supplementation significantly modulated the expression of *Wnt1* and *Ctnnb1* genes. CRC led to the downregulation of both genes, while OBG supplementation further decreased their expression, particularly at the highest concentration (3%). This illustrates the complex interplay between dietary factors and cancer status in affecting key signaling pathways involved in colorectal tumorigenesis. Additionally, WB and IHC results confirm that the effect of AOM-induced early stage of CRC is the downregulation of β-catenin protein expression in large intestine tissue. These results differ from the conventional understanding based on the literature, where the Wnt/β-catenin pathway is typically upregulated in intestinal cancer [65]. It is interesting to note that dietary factors like OBG might modulate this critical tumorigenic signaling pathway.

Carcinogenesis in the large intestine can lead to the loss of tumor suppressor genes, such as *APC* and *SMAD4*, which normally prevent cancer development [16]. On the other hand, during CRC development, the oncogene *Dclk1* is upregulated, promoting the proliferation of cancer stem cells, increasing tumor growth, and reducing sensitivity to conventional therapies. Therefore, *Dclk1* plays a crucial role in the progression of the disease [66]. In the present study, early-stage CRC significantly downregulates the tumor suppressor genes *Apc* and *Smad4*, reflecting their pivotal roles in cancer suppressive pathways. However, OBG supplementation mitigates this effect against the *Apc* gene, restoring its expression level closer to normal. The study demonstrates the potential of dietary interventions in modulating gene expression associated with cancer suppression. Specifically, the inclusion of 3% OBG in the diet restored Apc expression levels in CRC rats. This finding suggests that OBG supplementation may be a promising approach for cancer prevention. These results contribute valuable insights into the mechanistic pathways by which dietary components, such as beta-glucan, can influence the genetic transformations that occur during the development of colorectal cancer. This offers promising avenues for preventive strategies against tumorigenesis.

When investigating the impacts of dietary supplements on health, it is important to consider their long-term safety. It is worth noting that the consumption of 1-3, 1-4 beta glucan at 1% and 3% levels did not worsen systemic parameters or affect the clinical condition of the animals in the control groups, including fecal consistency. The addition of this polysaccharide to the feed did not affect its sensory qualities or growth performance. This is supported by the lack of differences in feed intake and equalized weight gains in all groups, including both CRC and controls. It is important to note that the OBG isolate has been shown to lack antioxidant activity. Therefore, it can be concluded that its effect on the redox status of CRC rats was indirect. This confirms that 1-3, 1-4 beta-glucan has a safe bioactivity profile. Additionally, its positive effects in pathological conditions, such as the early stages of colon cancer, make beta-glucan a promising candidate for consideration as a nutraceutical.

## 5. Conclusions

An *in vivo* study was conducted in a CRC model with a nutritional intervention that involved adding low molar mass oat beta-glucan at concentrations of 1% or 3% (*w*/*w*) to animal feed. The study showed the normalizing effects of this cereal polysaccharide. Particularly interesting were its effects at the 3% level on the redox status of peripheral blood and the signaling pathways associated with colon carcinogenesis. The precise extraction and purification process ensured the purity of OBG, which is crucial for accurately assessing its bioactive impact. This is particularly important in modulating genetic and biochemical pathways linked to tumorigenesis. The lack of adverse effects on the healthy intestine, combined with the strong beneficial effects of low molar mass oat beta-glucan in the early stages of carcinogenesis, underscores the safety and potent therapeutic impact of this compound. These observations support the consideration of this oat beta-glucan fraction as a promising nutraceutical. In summary, these findings highlight the potential of OBG as a dietary intervention to restore or maintain the balance of gene expressions crucial for preventing CRC progression.

## Figures and Tables

**Table 1:** Experimental groups.

	Control Groups (n = 21)			CRC Groups (n = 24)		
	OBG_0	OBG_1	OBG_3	OBG_0	OBG_1	OBG_3
Feed	Without OBG	+1% OBG	+3% OBG	Without OBG	+1% OBG	+3% OBG

Symbols indicate CRC—rats after AOM injection; OBG—low molar mass oat beta-glucan.

**Table 2:** Physicochemical characterization of low molar mass oat beta-glucan used as a feed additive (mean ± SE).

OBG	Molar Mass [g/mol]	Purity [%]	TPC [GAE/ 1 g s.m.]	DPPH [μmol TE/1 g s.m.]	Protein (%/db)	Nitrogen × 5.83 (%/s.m.)
Low molar mass	5.2 × 10^4^ ± 0.6 × 10^4^	99.3 ± 0.23	0.17 ± 0.040	5.94 ± 0.21	0.12 ± 0.04	1.52 ± 0.18

Symbols indicate TPC (Total phenolic compounds); GAE (Gallic acid equivalents); DPPH (2,2-diphenyl-1-picrylhydrazyl radical); TE (*Trolox equivalents*).

**Table 3:** Feed intake, feed growth efficiency, and total body weight gain (mean ± SE).

	Control Groups			CRC Groups		
	OBG_0	OBG_1	OBG_3	OBG_0	OBG_1	OBG_3
Feed intake [g/day]	25.84 ± 0.05	25.29 ± 0.44	25.48 ± 0.15	25.24 ± 0.33	24.98 ± 0.17	25.23 ± 0.33
Total body weight gain [g]	207.6 ± 3.57	196.1 ± 7.5	207.9 ± 10.58	205.1 ± 8.42	212.0 ± 5.82	199.1 ± 6.65
Feed growth efficiency [g/MJ] (mg/kcal)	9.006 ± 0.16 (38.65 ± 0.7)	8.842 ± 0.23 (37.68 ± 1.0)	9.644 ± 0.47 (40.53 ± 2.0)	9.107 ± 0.34 (39.09 ± 1.5)	9.691 ± 0.24 (41.29 ± 1.0)	9.342 ± 0.33 (39.26 ± 1.4)

The differences are not statistically significant.

**Table 4:** Large intestine epithelium changes.

	Control Groups			CRC Groups			χ^2^	*p*-Value/ V
	OBG_0 (n = 7)	OBG_1 (n = 7)	OBG_3 (n = 7)	OBG_0 (n = 8)	OBG_1 (n = 8)	OBG_3 (n = 8)		
colon epithelium changes	NC = 7 (100%)	NC = 7 (100%)	NC = 6 (86%) HP = 1 (14%)	NC = 3 (37.5%) HP = 5 (62.5%)	NC = 6 (75%) HP = 2 (25%)	NC = 7 (87.5%) HP = 1 (12.5%)	13.08	0.023/ 0.54

Symbols indicate HP—hyperplasia; NC—large intestine epithelium cells without histopathological changes; χ^2^—Pearson’s chi-square; V—Cramer’s V value.

**Table 5:** The length and width of intestinal crypts (mean ± SE).

Intestinal Crypts Parameters	Control Groups			CRC Groups		
	OBG_0	OBG_1	OBG_3	OBG_0	OBG_1	OBG_3
Crypt length [μm]	196.8 ± 10.23	196.4 ± 9.96	214.2 ± 9.10	220.5 ± 6.28	207.5 ± 8.20	223.5 ± 9.45
Crypt width bottom [μm]	33.41 ± 0.33	32.63 ± 0.52	32.88 ± 0.62	36.17 ± 1.33	34.21 ± 1.39	35.82 ± 0.69
Crypt width middle part [μm]	34.76 ± 1.28	34.37 ± 0.68	35.58 ± 0.87	34.02 ± 1.36	33.15 ± 0.97	34.88 ± 1.36
Crypt width upper part [μm]	37.39 ± 1.05	38.96 ± 1.32	39.96 ± 0.73	40.60 ± 1.69	38.51 ± 0.90	40.65 ± 1.09

The differences are not statistically significant.

**Table 6:** Erythrocyte parameters and platelet counts (mean ± SE).

Feature	Control Groups			CRC Groups		
	OBG_0	OBG_1	OBG_3	OBG_0	OBG_1	OBG_3
RBC [10^12^/L]	8.18 ± 0.29	8.01 ± 0.19	7.85 ± 0.28	8.07 ± 0.15	7.99 ± 0.17	8.09 ± 0.17
HBG [g/dL]	14.42 ± 0.26	14.23 ± 0.23	13.94 ± 0.42	14.45 ± 0.17	14.24 ± 0.32	14.50 ± 0.15
HCT [%]	42.52 ± 0.6	41.24 ± 0.56	41.03 ± 0.92	40.92 ± 0.84	39.92 ± 0.8	41.42 ± 0.59
MCV [fl]	53.00 ± 0.68	51.71 ± 0.87	51.83 ± 0.95	51.25 ± 0.67	50.00 ± 0.46	51.25 ± 0.80
MCH [pg]	17.64 ± 0.3	17.80 ± 0.25	17.77 ± 0.18	17.93 ± 0.21	17.80 ± 0.19	17.98 ± 0.28
MCHC [g/dL]	33.91 ± 0.2	34.49 ± 0.29	33.97 ± 0.43 ^A^	34.96 ± 0.24 *	35.31 ± 0.19 **	35.30 ± 0.22 **^, A^
RDWc [%]	17.59 ± 0.23	18.29 ± 0.24	17.98 ± 0.14	17.70 ± 0.21	18.04 ± 0.18	18.43 ± 0.19 *
PLT [10^9^/L]	718.7 ± 38.3	747.3 ± 32.2	788.9 ± 25.2	710.6 ± 22.7	781.4 ± 45.5	759.0 ± 36.0

OBG—low molar mass oat beta-glucan; RBC—red blood cells; HGB—hemoglobin; HCT—hematocrit; MCV—mean corpuscular volume; MCH—mean corpuscular hemoglobin; MCHC—mean corpuscular hemoglobin concentration; RDWc—red blood cell distribution width; PLT—platelet. The same letters denote significant differences between experimental groups determined by Tukey’s post hoc test (^A^ *p* < 0.05). Significant differences from the control group (control OBG_0) were determined by the Dunnett’s post hoc test (* *p* < 0.05, ** *p* < 0.01).

## Data Availability

The data that support the findings of this study are available on request from the corresponding author. The data are not publicly available due to privacy.

## Supplementary Materials

The following supporting information can be downloaded at: https://www.mdpi.com/article/10.3390/nu16081125/s1, Table S1: Product composition of AIN-93M experimental feeds declared by the producer; Table S2: Declared and analyzed composition of macronutrients (% *w*/*w*) in experimental feeds; Table S3: Results of histopathological examination of intestinal crypts; Supplementary Data: Microscope funding.

## References

[1] Nakashima A., Yamada K., Iwata O., Sugimoto R., Atsuji K., Ogawa T., Ishibashi-Ohgo N., Suzuki K. (2018). β-Glucan in foods and its physiological functions. J. Nutr. Sci. Vitaminol. (Tokyo).

[2] Wilczak J., Błaszczyk K., Kamola D., Gajewska M., Harasym J.P., Jałosińska M., Gudej S., Suchecka D., Oczkowski M., Gromadzka-Ostrowska J. (2015). The effect of low or high molecular weight oat beta-glucans on the inflammatory and oxidative stress status in the colon of rats with LPS-induced enteritis. Food Funct..

[3] Żyła E., Dziendzikowska K., Kamola D., Wilczak J., Sapierzyński R., Harasym J., Gromadzka-Ostrowska J. (2021). Anti-inflammatory activity of oat beta-glucans in a crohn’s disease model: Time- and molar mass-dependent effects. Int. J. Mol. Sci..

[4] Choromańska A., Kulbacka J., Harasym J., Oledzki R., Szewczyk A., Saczko J. (2017). High- and low-molecular weight oat beta-glucan reveals antitumor activity in human epithelial lung cancer. Pathol. Oncol. Res..

[5] Choromańska A., Kulbacka J., Rembiałkowska N., Piłat J., Olędzki R., Harasym J., Saczko J. (2015). Anticancer properties of low molecular weight oat beta-glucan—An in vitro study. Int. J. Biol. Macromol..

[6] Morgan E., Arnold M., Gini A., Lorenzoni V., Cabasag C.J., Laversanne M., Vignat J., Ferlay J., Murphy N., Bray F. (2023). Global burden of colorectal cancer in 2020 and 2040: Incidence and mortality estimates from GLOBOCAN. Gut.

[7] Jaroszyńska Z., Wiśniewska K. (2021). Epidemiology of colorectal cancer (C18-C21) in Poland. J. Educ. Health Sport.

[8] Shen J., Li P., Liu S., Liu Q., Li Y., Zhang Z., Yang C., Hu M., Sun Y., He C. (2020). The chemopreventive effects of Huangqin-tea against AOM-induced preneoplastic colonic aberrant crypt foci in rats and omics analysis. Food Funct..

[9] Sahebi Z., Emtyazjoo M., Mostafavi P.G., Bonakdar S. (2020). Promising Chemoprevention of Colonic Aberrant Crypt Foci by Portunus segnis Muscle and Shell Extracts in Azoxymethane-Induced Colorectal Cancer in Rats. Anticancer Agents Med. Chem..

[10] Clapper M.L., Chang W.C.L., Cooper H.S. (2020). Dysplastic Aberrant Crypt Foci: Biomarkers of Early Colorectal Neoplasia and Response to Preventive Intervention. Cancer Prev. Res..

[11] Ezuka A., Sakai E., Kawana K., Nagase H., Kakuta Y., Uchiyama S., Ohkubo H., Higurashi T., Nonaka T., Endo H. (2015). Association between factors associated with colorectal cancer and rectal aberrant crypt foci in humans. Oncol. Lett..

[12] Kobaek-Larsen M., Nielsen D.S., Kot W., Krych Ł., Christensen L.P., Baatrup G. (2018). Effect of the dietary polyacetylenes falcarinol and falcarindiol on the gut microbiota composition in a rat model of colorectal cancer. BMC Res. Notes.

[13] Madka V., Kumar G., Pathuri G., Zhang Y., Lightfoot S., Asch A.S., Mohammed A., Steele V.E., Rao C.V. (2020). Bisphosphonates zometa and fosamax synergize with metformin to prevent AOM-induced colon cancer in f344 rat model. Cancer Prev. Res..

[14] Perše M., Cerar A. (2011). Morphological and molecular alterations in 1,2 dimethylhydrazine and azoxymethane induced colon carcinogenesis in rats. J. Biomed. Biotechnol..

[15] Yang J., Wei H., Zhou Y., Szeto C.H., Li C., Lin Y., Coker O.O., Lau H.C.H., Chan A.W.H., Sung J.J.Y. (2022). High-Fat Diet Promotes Colorectal Tumorigenesis through Modulating Gut Microbiota and Metabolites. Gastroenterology.

[16] Chen J., Huang X.F. (2009). The signal pathways in azoxymethane-induced colon cancer and preventive implications. Cancer Biol. Ther..

[17] Im S.A., Kim J.W., Kim H.S., Park C.S., Shin E., Do S.G., Park Y.I., Lee C.K. (2016). Prevention of azoxymethane/dextran sodium sulfate-induced mouse colon carcinogenesis by processed Aloe vera gel. Int. Immunopharmacol..

[18] Saki E., Saiful Yazan L., Mohd Ali R., Ahmad Z. (2017). Chemopreventive Effects of Germinated Rough Rice Crude Extract in Inhibiting Azoxymethane-Induced Aberrant Crypt Foci Formation in Sprague-Dawley Rats. Biomed Res. Int..

[19] Raju J. (2008). Azoxymethane-induced rat aberrant crypt foci: Relevance in studying chemoprevention of colon cancer. World J. Gastroenterol..

[20] Harasym J., Suchecka D., Gromadzka-Ostrowska J. (2015). Effect of size reduction by freeze-milling on processing properties of beta-glucan oat bran. J. Cereal Sci..

[21] Harasym J., Żyła E., Dziendzikowska K., Gromadzka-Ostrowska J. (2019). Proteinaceous residue removal from oat β-glucan. Molecules.

[22] Harasym J., Olędzki R. (2018). The Mutual Correlation of Glucose, Starch, and Beta-Glucan Release During Microwave Heating and Antioxidant Activity of Oat Water Extracts. Food Bioprocess Technol..

[23] Harasym J., Satta E., Kaim U. (2020). Ultrasound treatment of buckwheat grains impacts important functional properties of resulting flour. Molecules.

[24] Kopiasz Ł., Dziendzikowska K., Oczkowski M., Harasym J., Gromadzka-Ostrowska J. (2024). Low-molar-mass oat beta-glucan impacts autophagy and apoptosis in early stages of induced colorectal carcinogenesis in rats. Int. J. Biol. Macromol..

[25] Reeves P.G., Nielsen F.H., Fahey G.C. (1993). AIN-93 purified diets for laboratory rodents: Final report of the American Institute of Nutrition ad hoc writing committee on the reformulation of the AIN-76A rodent diet. J. Nutr..

[26] Dell R.B., Holleran S., Ramakrishnan R. (2002). Sample size determination. ILAR J..

[27] Aguilar Diaz De Leon J., Borges C.R. (2020). Evaluation of Oxidative Stress in Biological Samples Using the Thiobarbituric Acid Reactive Substances Assay. JoVE.

[28] Nair A., Jacob S. (2016). A simple practice guide for dose conversion between animals and human. J. Basic Clin. Pharm..

[29] Bielohuby M., Bodendorf K., Brandstetter H., Bidlingmaier M., Kienzle E. (2010). Predicting metabolisable energy in commercial rat diets: Physiological fuel values may be misleading. Br. J. Nutr..

[30] Orlando F.A., Tan D., Baltodano J.D., Khoury T., Gibbs J.F., Hassid V.J., Ahmed B.H., Alrawi S.J. (2008). Aberrant crypt foci as precursors in colorectal cancer progression. J. Surg. Oncol..

[31] Velmurugan B., Singh R.P., Tyagi A., Agarwal R. (2008). Inhibition of azoxymethane-induced colonic aberrant crypt foci formation by silibinin in male Fisher 344 rats. Cancer Prev. Res..

[32] Suzuki R., Kohno H., Sugie S., Tanaka T. (2004). Sequential observations on the occurrence of preneoplastic and neoplastic lesions in mouse colon treated with azoxymethane and dextran sodium sulfate. Cancer Sci..

[33] Raju J., Swamy M.V., Cooma I., Patlolla J.M.R., Pittman B., Reddy B.S., Steele V.E., Rao C.V. (2005). Low doses of β-carotene and lutein inhibit AOM-induced rat colonic ACF formation but high doses augment ACF incidence. Int. J. Cancer.

[34] Kowalczyk M., Orłowski M., Klepacki Ł., Zinkiewicz K., Kurpiewski W., Kaczerska D., Pesta W., Zieliński E., Siermontowski P. (2020). Rectal aberrant crypt foci (ACF) as a predictor of benign and malignant neoplastic lesions in the large intestine. BMC Cancer.

[35] Corpet D.E., Taché S. (2002). Most Effective Colon Cancer Chemopreventive Agents in Rats: A Systematic Review of Aberrant Crypt Foci and Tumor Data, Ranked by Potency. Nutr. Cancer.

[36] Cheng L., Lai M. (2003). De Aberrant crypt foci as microscopic precursors of colorectal cancer. World J. Gastroenterol..

[37] Macharia J.M., Ngure V., Emődy B., Király B., Káposztás Z., Rozmann N., Erdélyi A., Raposa B. (2023). Pharmacotherapeutic Potential of *Aloe secundiflora* against Colorectal Cancer Growth and Proliferation. Pharmaceutics.

[38] Gigola G., Carriere P., Díaz M.B.N., Perdigon G., Zwenger A.O., Gentili C. (2021). Survival effect of probiotics in a rat model of colorectal cancer treated with capecitabine. World J. Gastrointest. Oncol..

[39] Bi W., Liu H., Shen J., Zhang L.H., Li P., Peng B., Cao L., Zhang P., He C., Xiao P. (2017). Chemopreventive effects of Ku-jin tea against AOM-induced precancerous colorectal lesions in rats and metabolomic analysis. Sci. Rep..

[40] Wang K.S., Yu G., Xu C., Meng X.H., Zhou J., Zheng C., Deng Z., Shang L., Liu R., Su S. (2021). Accurate diagnosis of colorectal cancer based on histopathology images using artificial intelligence. BMC Med..

[41] Wu R., Feng J., Yang Y., Dai C., Lu A., Li J., Liao Y., Xiang M., Huang Q., Wang D. (2017). Significance of Serum Total Oxidant/Antioxidant Status in Patients with Colorectal Cancer. PLoS ONE.

[42] Janion K., Szczepańska E., Nowakowska-zajdel E., Strzelczyk J., Copija A. (2020). Selected Oxidative Stress Markers in Colorectal Cancer Patients in Relation to Primary Tumor Location—A Preliminary Research. Medicina.

[43] Gu H., Li B., Xiang L., Xu Z., Tang Y., Zhu Z., Jiang Y., Peng L., He H., Wang Y. (2023). Association between oxidative stress exposure and colorectal cancer risk in 98,395 participants: Results from a prospective study. Front. Nutr..

[44] Acevedo-León D., Gómez-Abril S.Á., Sanz-García P., Estañ-Capell N., Bañuls C., Sáez G. (2023). The role of oxidative stress, tumor and inflammatory markers in colorectal cancer patients: A one-year follow-up study. Redox Biol..

[45] Wang J., Ding K., Wang Y., Yan T., Xu Y., Deng Z., Lin W., Zhang L., Zhu W., Zhao R. (2022). Wumei Pill Ameliorates AOM/DSS-Induced Colitis-Associated Colon Cancer through Inhibition of Inflammation and Oxidative Stress by Regulating S-Adenosylhomocysteine Hydrolase- (AHCY-) Mediated Hedgehog Signaling in Mice. Oxid. Med. Cell. Longev..

[46] Waly M.I., Ali A., Guizani N., Al-Rawahi A.S., Farooq S.A., Rahman M.S. (2012). Pomegranate (*Punica granatum*) peel extract efficacy as a dietary antioxidant against azoxymethane-induced colon cancer in rat. Asian Pac. J. Cancer Prev..

[47] Bardelčíková A., Šoltys J., Mojžiš J. (2023). Oxidative Stress, Inflammation and Colorectal Cancer: An Overview. Antioxidants.

[48] Liu H., Liu X., Zhang C., Zhu H., Xu Q., Bu Y., Lei Y. (2017). Redox Imbalance in the Development of Colorectal Cancer. J. Cancer.

[49] Moradi-Marjaneh R., Hassanian S.M., Mehramiz M., Rezayi M., Ferns G.A., Khazaei M., Avan A. (2019). Reactive oxygen species in colorectal cancer: The therapeutic impact and its potential roles in tumor progression via perturbation of cellular and physiological dysregulated pathways. J. Cell. Physiol..

[50] Lin S., Li Y., Zamyatnin A.A., Werner J., Bazhin A.V. (2018). Reactive oxygen species and colorectal cancer. J. Cell. Physiol..

[51] Basak D., Uddin M.N., Hancock J. (2020). The Role of Oxidative Stress and Its Counteractive Utility in Colorectal Cancer (CRC). Cancers.

[52] Malinowska K., Mik M., Dziki Ł., Dziki A., Majsterek I. (2015). Evaluation of antioxidant defense in patients with colorectal carcinoma. Pol. Prz. Chir. Pol. J. Surg..

[53] Kumar V.L., Verma S., Das P. (2019). Artesunate suppresses inflammation and oxidative stress in a rat model of colorectal cancer. Drug Dev. Res..

[54] Qi X., Liu Y. (2022). Anti-inflammatory and Antioxidant Effect of Lycoperoside H against the 1,2-Dimethyl Hydrazine (DMH) Induced Colorectal Cancer in Rats. J. Oleo Sci..

[55] Kopiasz Ł., Dziendzikowska K., Gajewska M., Wilczak J., Harasym J., Żyła E., Kamola D., Oczkowski M., Królikowski T., Gromadzka-Ostrowska J. (2020). Time-dependent indirect antioxidative effects of oat beta-glucans on peripheral blood parameters in the animal model of colon inflammation. Antioxidants.

[56] Wu J., Chen M., Shi S., Wang H., Li N., Su J., Liu R., Huang Z., Jin H., Ji X. (2017). Hypoglycemic effect and mechanism of a pectic polysaccharide with hexenuronic acid from the fruits of *Ficus pumila* L. in C57BL/KsJ db/db mice. Carbohydr. Polym..

[57] Sala R.J., Ery J., Cuesta-Peredo D., Muedra V., Rodilla V. (2023). Complete Blood Count Alterations Prior to the Diagnosis of Colorectal Cancer May Help in the Detection of Synchronous Liver Metastases. J. Clin. Med..

[58] Virdee P.S., Marian I.R., Mansouri A., Elhussein L., Kirtley S., Holt T., Birks J. (2020). The Full Blood Count Blood Test for Colorectal Cancer Detection: A Systematic Review, Meta-Analysis, and Critical Appraisal. Cancers.

[59] May J.E., Marques M.B., Reddy V.V.B., Gangaraju R. (2019). Three neglected numbers in the CBC: The RDW, MPV, and NRBC count. Cleve Clin. J. Med..

[60] Goyal H., Lippi G., Gjymishka A., John B., Chhabra R., May E. (2017). Prognostic significance of red blood cell distribution width in gastrointestinal disorders. World J. Gastroenterol..

[61] Wen Z.L., Zhou X., Xiao D.C. (2022). Is red blood cell distribution width a prognostic factor for colorectal cancer? A meta-analysis. Front. Surg..

[62] Waldman A.D., Fritz J.M., Lenardo M.J. (2020). A guide to cancer immunotherapy: From T cell basic science to clinical practice. Nat. Rev. Immunol..

[63] Laskowski T.J., Biederstädt A., Rezvani K. (2022). Natural killer cells in antitumour adoptive cell immunotherapy. Nat. Rev. Cancer.

[64] Cheng X., Xu X., Chen D., Zhao F., Wang W. (2019). Therapeutic potential of targeting the Wnt/β-catenin signaling pathway in colorectal cancer. Biomed. Pharmacother..

[65] Bian J., Dannappel M., Wan C., Firestein R. (2020). Transcriptional Regulation of Wnt/β-Catenin Pathway in Colorectal Cancer. Cells.

[66] Kalantari E., Razmi M., Tajik F., Asadi-Lari M., Ghods R., Madjd Z. (2022). Oncogenic functions and clinical significances of DCLK1 isoforms in colorectal cancer: A systematic review and meta-analysis. Cancer Cell Int..

