# Supplementary material for: Consumption of Feed Supplemented with Oat Beta-Glucan as a Chemopreventive Agent against Colon Cancerogenesis in Rats
Source: Nutrients. 2024 Apr 11;16(8):1125. doi: 10.3390/nu16081125 (PMC11054053; doi:10.3390/nu16081125)
Supplement: Supplementary file 1 [file nutrients-16-01125-s001.zip › Supplementary data - Microscope Funding.pdf]

We would like to acknowledge Regional Operational Programme of the Mazowieckie Voivodeship for purchase NIKON Eclipse Ti2 microscope. NIKON Eclipse Ti2 microscope was purchased as part of the “Food and Nutrition Centre – modernization of the WULS campus to create a Food and Nutrition Research and Development Centre (CŻiŻ)” co-financed by the European Union from the European Regional Development Fund under the Regional Operational Programme of the Mazowieckie Voivodeship for 2014–2020 (Project No. RPMA.01.01.00-14-8276/17).
