# Supplementary material for: Consumption of Feed Supplemented with Oat Beta-Glucan as a Chemopreventive Agent against Colon Cancerogenesis in Rats
Source: Nutrients. 2024 Apr 11;16(8):1125. doi: 10.3390/nu16081125 (PMC11054053; doi:10.3390/nu16081125)
Supplement: Supplementary file 1 [file nutrients-16-01125-s001.zip › Supplementary tables-nutrients-2920886.pdf]

Table S1. Product composition of AIN-93M experimental feeds declared by the producer (ZooLab, Sędziszów, Poland).

Ingredient (g/kg)	AIN-93M feed composition		
	OBG_0	OBG_1	OBG_3
Corn starch	467.49	456.49	436.49
Casein	140	140	140
Maltodextrin	155	155	155
Sucrose	100	100	100
Soybean oil	40	40	40
Fiber	50	50	50
Mineral mix (AIN-93g-MX)	35	35	35
Vitamin mix (AIN-93-VX)	10	10	10
Oat beta-glucan	0	11	31
Choline Hydrogen Tartrate	2.5	2.5	2.5
Tert-butylhydroquinone (TBHQ)	0.008	0.008	0.008

Table S2. Declared and analyzed composition of macronutrients (% w/w) in experimental feeds (for analyzed composition mean ± SD).

Nutrients in feeds	Declared composition			Analyzed Composition		
	OBG_0	OBG_1	OBG_3	OBG_0	OBG_1	OBG_3
Protein	14.0	14.0	14.0	12.86±1.79	12.73±1.8	12.84±1.80
Carbohydrates	72.25	71.15	69.15	75.9*	76.2*	75.6*
Fat	4.0	4.0	4.0	4.5±1.4	4.4±1.3	4.6±1.4
Fiber	5.0	5.0	5.0	3.8±0.5	4.0±0.7	4.1±0.7
beta-glucan	0.0	1.1	3.1	0.1 ± 0.02	1.06 ± 0.09	3.22 ± 0.56
Metabolizable energy [MJ/kg] (kcal/kg)	16.52 (3946,7)	16.22 (3873,4)	15.66 (3740,1)	–	–	–

* The carbohydrates content was calculated from the difference based on the content of water, protein, fat and ash

Table S3. Results of histopathological examination of intestinal crypts.

Rat number	Grup	Feed	Results of histopathological examination of intestinal crypts.
**39**	Control	OBG_0	No changes in crypt epithelial cells
**40**	Control	OBG_0	No changes in crypt epithelial cells
**41**	Control	OBG_0	No changes in crypt epithelial cells
**42**	Control	OBG_0	No changes in crypt epithelial cells
**43**	Control	OBG_0	No changes in crypt epithelial cells

**44**	Control	OBG_0	No changes in crypt epithelial cells
**45**	Control	OBG_0	No changes in crypt epithelial cells
**25**	Control	OBG_1	No changes in crypt epithelial cells
**26**	Control	OBG_1	No changes in crypt epithelial cells
**27**	Control	OBG_1	No changes in crypt epithelial cells
**28**	Control	OBG_1	No changes in crypt epithelial cells
**29**	Control	OBG_1	No changes in crypt epithelial cells
**30**	Control	OBG_1	No changes in crypt epithelial cells
**31**	Control	OBG_1	No changes in crypt epithelial cells
**32**	Control	OBG_3	No changes in crypt epithelial cells
**33**	Control	OBG_3	No changes in crypt epithelial cells
**34**	Control	OBG_3	No changes in crypt epithelial cells
**35**	Control	OBG_3	No changes in crypt epithelial cells
**36**	Control	OBG_3	**Hyperplasia**
**37**	Control	OBG_3	No changes in crypt epithelial cells
**38**	Control	OBG_3	No changes in crypt epithelial cells
**17**	CRC	OBG_0	**Hyperplasia**
**18**	CRC	OBG_0	**Hyperplasia**
**19**	CRC	OBG_0	**Hyperplasia**
**20**	CRC	OBG_0	No changes in crypt epithelial cells
**21**	CRC	OBG_0	No changes in crypt epithelial cells
**22**	CRC	OBG_0	No changes in crypt epithelial cells
**23**	CRC	OBG_0	**Hyperplasia**
**24**	CRC	OBG_0	**Hyperplasia**
**1**	CRC	OBG_1	**Hyperplasia**
**2**	CRC	OBG_1	No changes in crypt epithelial cells
**3**	CRC	OBG_1	No changes in crypt epithelial cells
**4**	CRC	OBG_1	No changes in crypt epithelial cells
**5**	CRC	OBG_1	No changes in crypt epithelial cells
**6**	CRC	OBG_1	**Hyperplasia**
**7**	CRC	OBG_1	No changes in crypt epithelial cells
**8**	CRC	OBG_1	No changes in crypt epithelial cells
**9**	CRC	OBG_3	No changes in crypt epithelial cells
**10**	CRC	OBG_3	No changes in crypt epithelial cells
**11**	CRC	OBG_3	No changes in crypt epithelial cells
**12**	CRC	OBG_3	No changes in crypt epithelial cells
**13**	CRC	OBG_3	No changes in crypt epithelial cells
**14**	CRC	OBG_3	**Hyperplasia**
**15**	CRC	OBG_3	No changes in crypt epithelial cells
**16**	CRC	OBG_3	No changes in crypt epithelial cells
